# Understanding and Addressing Occupational Stressors in Internet-Delivered Therapy for Public Safety Personnel: A Qualitative Analysis

**DOI:** 10.3390/ijerph19084744

**Published:** 2022-04-14

**Authors:** Janine D. Beahm, Caeleigh A. Landry, Hugh C. McCall, R. Nicholas Carleton, Heather D. Hadjistavropoulos

**Affiliations:** 1 Department of Psychology, University of Regina, 3737 Wascana Pkwy, Regina, SK S4S 0A2, Canada; janine.beahm@uregina.ca (J.D.B.); caeleigh.landry@uregina.ca (C.A.L.); hugh.mccall@uregina.ca (H.C.M.); nick.carleton@uregina.ca (R.N.C.); 2 PSPNET, University of Regina, 2 Research Drive, Regina, SK S4T 2P7, Canada

**Keywords:** public safety personnel, first responder, internet-delivered cognitive behavioral therapy, digital mental health, occupational stress

## Abstract

Internet-delivered cognitive behavioral therapy (ICBT) is effective when tailored to meet the needs of public safety personnel (PSP). Nevertheless, there is limited research on the nature of the occupational stressors faced by PSP who seek ICBT and how PSP use ICBT to address occupational stressors. We provided tailored ICBT to PSP (*N* = 126; 54% women) and conducted a qualitative content analysis on clinicians’ eligibility screening notes, clients’ emails, and clients’ survey responses to understand the occupational stressors faced by PSP and their use of ICBT to address such stressors. Clients described several occupational stressors, including operational stressors (e.g., potentially psychologically traumatic events and sleep/shiftwork issues) and organizational stressors (e.g., issues with leadership, resources, and workload). More clients shared occupational concerns during the screening process (97%) than during treatment (58%). The most frequently cited occupational stressor was exposure to potentially psychologically traumatic events. Clients reported using course skills (e.g., controlled breathing and graduated exposure) to manage occupational stressors (e.g., responding to calls, workplace conflict, and work–family conflict). Thought challenging was the most frequently reported strategy used to manage occupational stressors. The current results provide insights into the occupational stressors PSP experience and endeavor to manage using ICBT, which can inform further efforts to tailor ICBT for PSP (e.g., adapting course materials and examples to take into account these operational and occupational stressors).

## 1. Introduction

Public safety personnel (PSP) refers to diverse professionals working to keep communities safe. PSP include, but are not limited to, border services officers, correctional workers, firefighters (career and volunteer), Indigenous emergency managers, operational intelligence personnel, paramedics, police (municipal, provincial, and federal), public safety communicators, and search and rescue personnel [1]. Elevated rates of mental health challenges have been observed among PSP worldwide, including police [2], paramedics [3], firefighters [4], and disaster and rescue workers [5,6]. Among a large sample of diverse Canadian PSP (*N* = 5813), 44.5% screened positive for clinically significant symptoms of one or more mental health disorders [7]. Symptoms were found to be particularly elevated among PSP who were female, were unmarried, or had not completed a university or college degree. Since 2015, the Canadian government has increasingly focused attention on the high rates of mental health disorder symptoms associated with posttraumatic stress injuries among PSP, as well as the need for accessible and effective treatments [8]. Despite the increased attention and increased availability of mental health training programs, PSP continue to report logistical barriers to care (e.g., geographical and time), concerns about therapists not understanding them, and concerns about confidentiality [9]. PSP also continue to report experiencing significant and pervasive workplace stigma related to mental health [10,11].

Occupational stressors that may contribute to PSP mental health concerns have been categorized into two domains: (1) operational stressors (i.e., related to job duties); and (2) organizational stressors (i.e., related to job context) [12]. Operational stressors for PSP include exposure to potentially psychologically traumatic events, sleep disturbances, chronic pain and injuries, fatigue from shift work, increased vigilance, work location, conflicts between work and personal life, and public scrutiny [12,13]. Organizational stressors for PSP include interpersonal dynamics, lack of support from management, workplace bullying, stress related to job promotions, staff shortages, and a lack of resources resulting in unmanageable workloads [12]. PSP describe their occupational stressors as exacerbated by the PSP organizational culture, which typically reflects a paramilitary and hierarchical reporting structure [14]. Furthermore, there is growing evidence that PSP mental health may be just as impacted by organizational stressors as operational stressors [12] and that organizational stressors can amplify the impacts of operational stressors (e.g., PPTE exposures) [14]. Occupational stressor experiences can vary by PSP sector [12], gender [15], and employment location [16].

Internet-delivered cognitive behavioral therapy (ICBT) is a digital mental health intervention that is based on the concepts of cognitive behavioral therapy and overcomes barriers to care by providing online services through secure encryption technology that supports confidentiality and accessibility [17]. There are more than two decades of research evidence demonstrating ICBT as effective for treating various mental health disorder symptoms, with treatment outcomes comparable to face-to-face therapy when paired with therapist support [17,18,19,20]. ICBT interventions can be designed to treat symptoms of a specific mental health disorder (i.e., disorder-specific) or designed to treat symptoms of several disorders (i.e., transdiagnostic). Results from survey and interview research suggest Canadian PSP view ICBT as a valued form of mental health treatment [9,21].

The 2019 Government of Canada National Action Plan for Addressing Post Traumatic Stress Injuries described the potential value of ICBT for PSP, leading to funding for the development and delivery of ICBT tailored for PSP [15]. The ICBT tailored for PSP is called PSPNET, and currently offers therapist-assisted ICBT to PSP residing in Saskatchewan, Quebec, Nova Scotia, New Brunswick, and Prince Edward Island, with intentions to expand to other provinces. PSPNET currently offers two treatment courses: the transdiagnostic *PSP Wellbeing Course* and the disorder-specific *PSP Posttraumatic Stress Disorder (PTSD) Course*. A self-guided version of the *PSP Wellbeing Course* is currently offered to PSP anywhere in Canada. PSPNET courses are the first ICBT courses tailored specifically for PSP. Initial outcomes from the first 83 PSP enrolled in the *PSP Wellbeing Course* in Saskatchewan showed large reductions in symptoms of anxiety and depression, moderate reductions in symptoms of PTSD, and small reductions in symptoms of social anxiety [22]. Qualitative data collected from clients during the first six months of enrolment also showed that the majority of clients were satisfied with the course and found it beneficial [23]. Given that this is the first ICBT program to address PSP, it is not currently known how PSP are using ICBT to manage occupational stressors.

The goal of the current study is to examine the occupational stressors PSP describe when seeking and receiving ICBT and how they use skills learned in ICBT to manage those stressors. Specifically, the current study was designed to: (1) identify the nature and scope of occupational stressors that PSP report when initially seeking ICBT; (2) explore the nature and scope of occupational stressors that PSP share while enrolled in ICBT; (3) explore whether the occupational stressors impacting PSP mental health vary by occupation, gender, community size (i.e., located in a rural versus urban area), and symptoms of mental disorders (i.e., clinically significant versus non-clinically significant); and (4) identify the extent to which PSP describe various ICBT skills as helpful for managing occupational stressors. The study uses qualitative data that are appropriate for exploring client experiences within digital mental health programs [24], describing occupational stressors [25], and guiding enhancements to the quality of interventions [26].

## 2. Materials and Methods

### 2.1. Context

The current study collected qualitative data from clients who enrolled in the *PSP Wellbeing Course*, a transdiagnostic ICBT course tailored by PSPNET to meet the unique needs of Canadian PSP. The original *Wellbeing Course* was developed by the eCentre Clinic at Macquarie University, Australia, and has been implemented and evaluated in Australia and Saskatchewan, Canada, with evidenced effectiveness for reducing symptoms of anxiety-, mood-, and trauma-related disorders [27,28,29,30,31,32,33,34,35]. The PSPNET team began tailoring the *Wellbeing Course* by conducting interviews with 126 PSP stakeholders in Saskatchewan and Quebec, which supported the initial adaptations [9]. The PSPNET team continues to make iterative improvements to the course based on client feedback [23]. The current study is part of a registered observational trial of the *PSP Wellbeing Course* (Clinical Trials.gov NCT04127032), which was approved by the institutional research ethics board at the University of Regina (REB#: 2019-157).

### 2.2. Course Description and Eligibility Criteria

**Figure 1:**
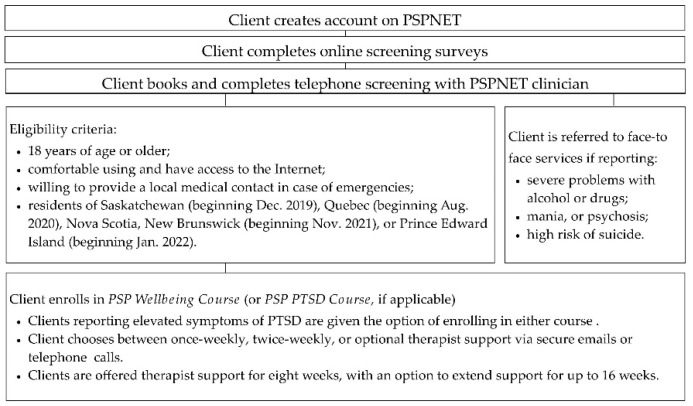
Process for enrolling in and completing the *PSP Wellbeing Course*.

**Figure 2:**
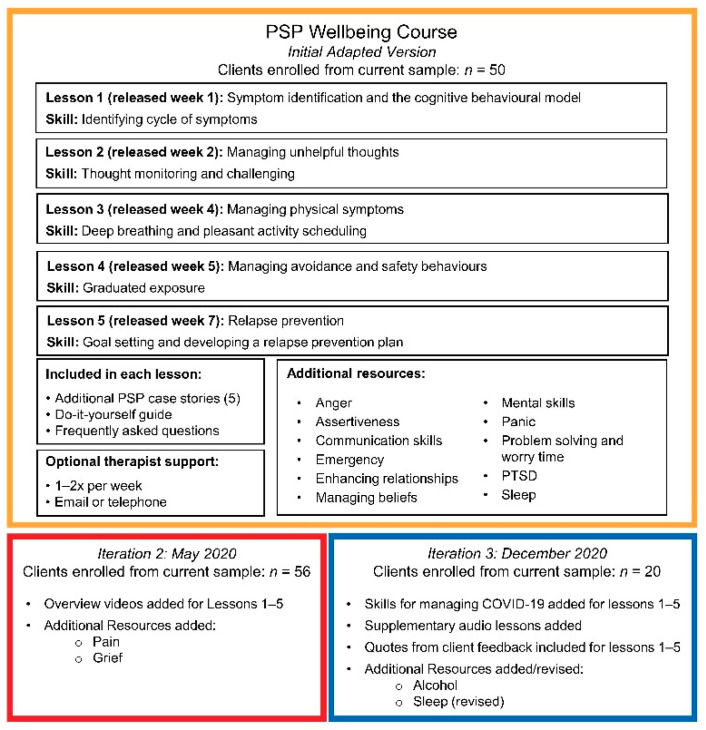
Description of the *PSP Wellbeing Course*.

The eligibility screening and enrollment processes for the *PSP Wellbeing Course* (offered in English and French) are presented in Figure 1. The five main psychoeducational lessons and other content (including content in the original version of the course and content subsequently added) are presented in Figure 2. Making such changes to ICBT interventions during clinical trials is consistent with recommendations on implementing ICBT and other digital mental health interventions [36]. Course lessons are gradually released according to an 8-week timeline. Clients can access optional therapist support for up to 16 weeks and can access lesson slides, as well as downloadable and printable additional resources and DIY guides for up to 1 year after enrollment (course slides cannot be downloaded).

### 2.3. Participants

The current study includes data from 126 clients who were enrolled in the English version of the *PSP Wellbeing Course* in Saskatchewan between 5 December 2019 and 15 March 2021. Clients who officially withdrew from the program (*n* = 17) were excluded from the analyses to address concerns regarding confidentiality or limited data. The sample size was adequate for the study design [37]. A list of client characteristics, including demographics and mental disorder symptoms at enrollment, can be found in Table 1.

### 2.4. Measures and Data

Clients completed the Patient Health Questionnaire-9 (PHQ-9; [38]), Generalized Anxiety Disorder-7 (GAD-7; [39]), and PTSD Checklist for DSM-5 (PCL-5; [40]) during the online screening. Client survey data were used to describe symptom levels at enrollment. After completing the surveys, clients scheduled and completed a telephone interview with a therapist to assess their course eligibility. Therapists compiled screening notes for each client based on the client’s responses to the online surveys, as well as additional information provided during the telephone interview. The notes were used to summarize clients’ self-reported symptoms and associated major contributing factors. Once enrolled in the course, clients were encouraged to complete weekly symptom and reflection surveys, as well as a Treatment Satisfaction Questionnaire (TSQ; see Appendix A) administered at 8-weeks post-enrollment (available to complete for up to 4 weeks). The weekly surveys include questions assessing challenges with the course materials, inviting examples of completed course work, helpful elements of coursework, and elements that need improving. Clients were regularly encouraged to exchange emails or schedule telephone calls with their therapist. The current study data included therapist screening notes, open-ended emails to therapists, responses to weekly surveys, and TSQ responses. All client emails sent within 16 weeks of enrollment in the course were included in the analyses. Additional questionnaires not germane to the purposes of the present study are described elsewhere [22].

### 2.5. Analysis

All data sources for each client were de-identified and compiled into a single client file. Each client case file was input into the qualitative analysis software NVIVO 12.0 [41]. Client demographic data were entered for each client case (i.e., PSP occupation, gender, race/ethnicity, community size, and symptoms of mental disorders). The data were coded using a qualitative inductive content analysis approach [42]. The screening data were coded to identify the nature and scope of occupational stressors PSP hope to manage with ICBT (objective one). Data were coded to explore occupational stressors (objective two) and the skills PSP describe as helpful for managing occupational stressors (objective four). Clients could endorse more than one category or subcategory. Categories were further grouped under larger domains. A crosstab query with all coded data was run using NVIVO 12.0 to assess for demographic covariates (objective three). The query produced main domains and categories of interest including occupation, gender, location of work (i.e., urban or non-urban), and symptoms of mental disorders (i.e., clinically significant versus non-clinically significant). There was insufficient diversity within the sample to assess for race and ethnicity in the query.

The data were initially coded into domains, categories, and subcategories. The coding scheme was then discussed and refined through conversations with the PSPNET research team until all disagreements were resolved. Response frequencies are reported to emphasize overall trends of commonly endorsed categories. Summarized examples of text coded within each category are included to describe the categories. Client quotations are not used because of data sensitivity and confidentiality considerations.

## 3. Results

### 3.1. Client Course Usage and Completion Rates

Client course usage and lessons accessed are summarized in Table 2.

### 3.2. Occupational versus Personal Stressors Reported during Eligibility Screen

Most clients reported experiencing one or more impactful occupational stressors (*n* = 122/126; 96.8%). Operational stressors (*n* = 113, 89.6%) were endorsed more frequently than organizational stressors (*n* = 57, 45.2%). The most frequently cited occupational stressor overall was exposure to PPTEs (*n* = 100, 79.3%). Some clients reported PPTE exposures, but described them as having a limited impact on their mental health or described other stressors as more impactful (*n* = 21, 16.7%). Many clients (*n* = 57, 45.2%) reported that organizational stressors were contributing to their symptoms, with some (*n* = 10, 7.9%) specifically commenting on interactions with operational stressors. Clients commented on how under-resourcing increases the risk and frequency of PPTEs and leaves PSP unable to meet public expectations, have debriefing time, and take even brief respites between PPTEs. Most clients (*n* = 83, 65.9%) also reported seeking ICBT because of personal stressors or concerns outside of occupational stressors. Some clients (*n* = 10, 7.9%) reported taking the *PSP Wellbeing Course* as a proactive or educational measure and reported few-to-no symptoms during screening. Table 3 provides details on the types of occupational and personal stressors that PSP reported during their screen. Supplementary Tables S1 and S2 delineate occupational stressors reported during the screen by gender, PSP occupation, location of work, and clinically significant symptoms of mental disorders (i.e., anxiety, depression, and post-traumatic stress).

### 3.3. Occupational and Personal Issues Shared with Therapists

PSP shared concerns and stressors with their therapists to ask for general advice, to ask for suggestions about how to deal with situations, or to use a safe space to share their concerns (see Table 4). PSP described occupational stressors (*n =* 73, 57.9%) and personal stressors (*n* = 49, 38.7%). The frequency of occupational stressors was slightly higher than that of personal stressors. Supplementary Tables S3 and S4 provide a breakdown of occupational stressors by gender, PSP occupation, location of work, and clinically significant symptoms of mental disorders.

### 3.4. Use of Skills and Resources for Managing Occupational and Personal Stressors

PSP described several skills from the course as helpful, but only some provided specific context; for example, clients described the skills as helpful for managing stress related to PPTE exposures, dealing with demanding or combative clients or patients, attending trial, managing flashbacks or symptoms related to previous calls, managing feelings of inadequacy related to their job, becoming more social at work, or being more assertive about harassment situations at work. Clients also described the skills as helpful for improving personal relationships impacted by their work (e.g., such as managing anger and outbursts towards family). Clients made relatively few comments about using the skills in their personal lives without also providing a work-related context. Table 4 presents the number of clients who reported finding each skill helpful, and the number of clients who reported either finding the skill helpful or working on using each skill, delineated by context (i.e., in a work-related context, personal context, or unspecified context).

Thought challenging was the most frequently cited skill that clients were using and finding helpful; paradoxically, clients also reported having challenges with using thought challenging more frequently than with any other skill. Table 5 shows the number of clients who reported experiencing challenges with each skill. Challenges included the time required to practice and implement each skill, not understanding how to implement the skill, seeking advice on how to implement the skill, and difficulties overcoming previous patterns when implementing the skill (e.g., stopping previous negative thoughts). Table 6 presents a summary of additional resources clients used or found helpful, as well as challenges associated with the resources.

Many clients (*n* = 47, 37.3%) reported PSP-specific case stories and examples were helpful; however, some clients (*n* = 15, 11.9%) reported not liking or not resonating with the case stories. There were 15 clients who were not fully satisfied with the stories: 2 reported not liking reading stories in general; 2 reported not finding the stories helpful, but believing other people might find the stories helpful; 1 reported liking the stories, but wanting additional examples; 1 reported identifying with some aspects of the examples, but not others; 2 reported that the stories were unrealistic and contrived; and 7 did not provide further explanation.

## 4. Discussion

PSP experience high rates of mental health challenges, which have been associated with several operational and organizational stressors [12]. PSPNET provides the first ICBT program tailored to meet the needs of PSP. The current study explored how PSP occupational issues present within an ICBT clinic. Specifically, the study examined the scope and nature of occupational stressors experienced by a sample of PSP who accessed an ICBT course (the *PSP Wellbeing Course*) and how participating PSP sought and used the course to cope with their occupational stressors.

### 4.1. Primary Results

Clients reported using ICBT to manage occupational stressors at a higher frequency than they did to manage personal stressors. Almost all clients who sought ICBT reported an occupational stressor was impacting their mental health during the screening process. PSP also reported personal stressors as reasons for seeking ICBT, but at a lower frequency than occupational stressors. Operational stressors were the most frequently reported stressor and included exposure to PPTEs, sleep and shiftwork issues, hypervigilance, pain or injury, and issues with the public. PPTEs were the most frequently cited operational stressor. Organizational stressors were also reported and included issues with administration, leadership, or management, resources and workload, issues with co-workers, and complaints. About half of clients reported other factors impacting their mental health, including the impact their work had on their family life, organizational stressors, and the impacts of COVID-19. About a quarter of clients also cited unspecified work-related stressors as affecting their mental health. The current results reflect previous research results that underscore the mental health impact of operational and organizational stressors for PSP [12], as well as emphasizing the impact PSP work can have on families, which can reciprocally impact the mental health of PSP [43]. The current results also highlight the impact of the COVID-19 pandemic on Saskatchewan PSP [44].

Reported frequencies were framed by the screening questions, which included a PPTE survey, a sleep survey, questions on vigilance, and a survey on the impacts of COVID-19, but we did not assess for other operational or organizational stressors or assess the impact of PSP work on family life. Most questions focused on operational stressors, rather than organizational stressors; accordingly, cuing may explain some of the operational stressors being highlighted more often than organizational stressors in the screening notes. When sharing concerns with their therapists, clients discussed operational and organizational concerns at similar frequencies. Prior research suggests that organizational stressors may substantially impact PSP mental health [12] and clients in the current study reported using ICBT to manage both operational and organizational stressors.

During the eligibility screening process, approximately half of all clients reported their work impacted their family life, highlighting the significant impact PSP work can have on their families. Most clients cited learning to manage occupational stressors as a way to minimize the impact of their work on their families, and a reason for wanting to engage with the *PSP Wellbeing Course*. The mitigation rationale may explain why PSP focused on wanting to help their families in the screening, but focused on managing occupational stressors when discussing concerns with therapists. In any case, the current results underscore the need to address the impact of PSP work on families.

Most client comments coded under the domain skill use did not include specific contexts. Where context was provided, clients reported using the course skills to manage occupational stressors more often than personal stressors. Clients who provided specific context for skill use to address occupational stressors suggested the skills helped to manage operational (e.g., PPTEs, emergency calls, and attending trial) and organizational (e.g., socializing with co-workers and harassment) stressors, suggesting that ICBT can provide PSP with skills for managing several occupational stressors. Thought challenging was the most frequently used skill overall, which is consistent with previous research [23], highlighting thought challenging as specifically helpful for occupational and personal stressors. However, the popularity of thought challenging could be partially explained by the fact that it was one of the first skills taught in the *PSP Wellbeing Course*, and nearly all clients learned about it, while some clients did not progress far enough in the course to learn some of the skills taught in later lessons. Nevertheless, these findings suggest that ICBT tailored for PSP should emphasize thought challenging and provide sufficient examples of how it can be used to manage occupational stressors. This implication may also extend to face-to-face cognitive behavioral therapy.

Previous research on the *PSP Wellbeing Course* evidenced promising outcomes for symptom improvement [22], with almost all clients benefitting from the course [23]. The current data also support the *PSP Wellbeing Course* as beneficial for managing mental health disorder symptoms related to current or previous occupational stressors, as well as other personal stressors. Clients shared concerns about occupational stressors with their therapists more frequently than they reported specific skill use in the context of occupational stressors; clients appeared to be using therapist support to seek feedback or support for their thoughts and feelings more often than to discuss course skill applications. The results appear consistent with previous research indicating ICBT therapist interactions involve much more than simple program engagement [45]. The broad evidence supports the *PSP Wellbeing Course* with therapist interactions as beneficial for treating diverse PSP mental health challenges [22,23].

Comparisons of occupational stressors across PSP sectors, gender, and location of employment evidenced very few between-group differences; however, the absent differences may be due to client decisions about sharing information rather than an actual difference in experiences. The current results align with previous research results, suggesting that occupational stressors are pervasive across gender and PSP sectors [14]. Detailed analyses of stressors experienced within each theme remain outside the scope of the present study. Future research may identify important nuances in experiences of operational stressors, such as specific gender differences in issues with management, work and family life balance [15,46], or differences between urban, rural, and remote service locations [16].

Comparisons of occupational stressors by clients who were experiencing clinically significant versus non-clinically significant symptoms of mental disorders (i.e., anxiety, depression, and post-traumatic stress) showed that clients who had clinically significant symptoms may be more likely to present occupational stressors as a reason for seeking ICBT and to discuss occupational stressors within ICBT. However, clients who were not experiencing clinically significant symptoms also used ICBT to manage occupational issues. This suggests ICBT may be helpful for PSP without clinically significant symptoms as a proactive measure for managing occupational stressors.

### 4.2. Application of Findings

The current study results inform the iterative changes to the *PSP Wellbeing Course* as part of a learning health system framework designed to rapidly integrate research results into practice [47,48,49], which is considered best practice for ICBT and other digital mental health interventions during clinical trials [36,50,51,52]. A learning health system approach supports following successive iterations of learning cycles that results in research data informing practice revisions, and practice revisions informing subsequent research [47,53]. The current data informed the optimization of the course. New resources were added to the *PSP Wellbeing Course* to provide more comprehensive supports based on the experiences clients shared with their therapists, for example, (1) a health anxiety resource was added for PSP experiencing anxiety related to contracting COVID-19; (2) a resource for families was developed and added based on PSP comments that their work was impacting their family life and that PSP were using the *PSP Wellbeing Course* with the support of a significant other; and (3) a resource on seeking mental health supports in the workplace was developed and added based on client concerns about lack of support from management.

Most clients reported finding the stories helpful, but some clients provided feedback on how the stories could be improved. Persuasive design is a set of design principles intended to promote engagement with technology [54] that can produce greater symptom reduction from ICBT [55]. A persuasive design principle suggests user motivations for specific actions increase when observing others doing the same actions and benefiting from the behaviors [54]. The use of case studies or patient stories in health interventions are typically well accepted and are often described by clients as helpful [56,57]. Qualitative and quantitative evidence suggests case stories can provide information, model behavior, increase knowledge and confidence, improve personal health, and provide a sense of comfort [58]. Benefits from case studies or patient stories appear mediated by user affinity, suggesting interventions need to incorporate information patients perceive as relevant and accurate [59]. Therefore, the results were also used to adapt case stories, such as making technical changes to descriptions of occupational duties and changes to examples of the use of course skills (e.g., thought challenging).

The findings from the current study can also be used by other digital mental health providers seeking to treat PSP. Worldwide, PSP face unique occupational stressors, which lead to increased rates of mental disorders within these populations [5]. Given that PSPNET is the first ICBT program tailored to meet the needs of PSP, this study informs about the occupational stressors that are likely to present in ICBT. This information can be used to optimize course materials and examples to assist PSP in managing occupational stressors.

### 4.3. Limitations and Future Research

The data are limited to what the PSP shared with their therapists through email conversations, weekly reflections, the TSQ, and during the eligibility screen. Not all PSP shared their experiences with their therapists through emails. There are data for all screens; however, some PSP may have also been experiencing other occupational stressors without reporting the stressors to the therapists. Reported frequencies within screening data were also likely affected by the content of the questions asked during the screening process. The data cannot be used to infer specific rates at which PSP use ICBT to manage types of occupational stressors, but can provide insight into general patterns and trends [60]. The sample is relatively homogenous such that not all PSP sectors were comparably represented and some important occupational stressors may not have been reported. The current sample sizes did not allow for comparisons of occupational stressors by race or ethnicity. Future research may examine if there is a relationship between client characteristics, personal stressors, and occupational stressors.

## 5. Conclusions

The current study explored the occupational stressors PSP describe when seeking and receiving ICBT. It also showed how PSP apply skills learned in ICBT to manage both occupational and personal stressors. Occupational stressors included diverse operational and organizational stressors. The current study also evidenced that PSP use several cognitive behavioral therapy skills to manage occupational stressors. The cognitive behavioral therapy skill “thought challenging” was cited as the most helpful for managing occupational stressors. The results provide insight into PSP experiences of occupational stressors, the impact of occupational stressors on PSP mental health, and how PSP use ICBT to manage occupational stressors. The current results can guide the further tailoring of ICBT to PSP and can help to prepare clinicians who provide mental health services for PSP by informing them about the types of examples to include and the skills that PSP find helpful for managing stressors.

## Figures and Tables

**Table 1:** Client characteristics.

Characteristics	Total Sample (*N* = 126)
**Gender, *n* (%)**	
Man	57 (45)
Woman	68 (54)
Nonbinary	*
**Community size, *n* (%)**	
<100,000 (non-urban)	62 (49)
>100,000 (urban)	64 (51)
**Relationship status, *n* (%)**	
Not married or common law	45 (36)
Married or common law	81 (64)
**Race/ethnicity, *n* (%)**	
White	111 (88)
First Nations, Inuit, or Métis	11 (9)
Other ethnic minorities	*
**Years of experience in PSP occupation**	
Mean (*SD*)	12 (8.2)
0–9 years, *n* (%)	48 (38)
10+ years, *n* (%)	77 (61)
No response, *n* (%)	*
**Highest level of education, *n* (%)**	
No degree	55 (44)
College diploma	34 (27)
University degree	36 (29)
**Age**	
Mean (*SD*)	40.7 (10.4)
19–29, *n* (%)	22 (18)
30–39, *n* (%)	35 (28)
40–49, *n* (%)	41 (33)
50–59, *n* (%)	23 (18)
60+, *n* (%)	5 (4)
**PSP occupation, *n* (%)**	
Border Services	5 (4)
Corrections	20 (16)
Dispatch/Communications	8 (6)
Fire	9 (7)
Paramedicine	38 (30)
Police	36 (29)
Other (e.g., nurse and peace officer)	10 (8)
**Treatments within past three months, *n* (%)**	
Taken mental health medication	40 (32)
Seen mental healthcare provider	64 (51)
**Pre-treatment PHQ-9**	
Mean (*SD*)	11.5 (6.0)
Not clinically significant (0–9), *n* (%)	51 (40)
Clinically significant (10–27), *n* (%)	75 (60)
**Pre-treatment GAD-7**	
Mean (*SD*)	10.4 (5.7)
Not clinically significant (0–9), *n* (%)	57 (45)
Clinically significant (10–21), *n* (%)	67 (53)
No response, *n* (%)	*
**Pre-treatment PCL-5**	
Mean (*SD*)	29.3 (18.6)
Not clinically significant (0–32), *n* (%)	75 (60)
Clinically significant (33–80), *n* (%)	51 (40)

* Indicates there were at least 1, but fewer than 5, clients in the grouping. The exact number was masked to protect confidentiality.

**Table 2:** Client course usage and lesson completion rates.

Course Usage and Lessons Accessed	Total Sample (*N* = 126)
**Lessons accessed at 8 weeks, *n* (%)**	
Lesson 1	124 (98)
Lesson 2	118 (94)
Lesson 3	108 (86)
Lesson 4	95 (75)
Lesson 5	77 (61)
**Lessons accessed at 16 weeks, *n* (%)**	
Lesson 1	125 (99)
Lesson 2	119 (94)
Lesson 3	111 (88)
Lesson 4	103 (82)
Lesson 5	96 (76)
**Number of emails sent**	
Mean (*SD*)	5.5 (5.1)
0–9, *n* (%)	25 (20)
10–19, *n* (%)	87 (69)
20+, *n* (%)	13 (10)
**Number of telephone calls**	
Mean (*SD*)	2 (3.0)
0–4, *n* (%)	105 (83)
5–10, *n* (%)	17 (14)
10+, *n* (%)	4 (3)
**Completed Treatment Satisfaction Questionnaire**	
Yes	109 (87)
No	17 (14)

**Table 3:** Reasons for seeking ICBT, including occupation and personal stressors, reported during screening (*N* = 126).

Domain/Category/Subcategory	Definition	Examples	*n* (%)
**Occupational Stressors**	Amalgamation of occupational stressors categories and subcategories.		122 (97)
(1) Operational Stressors	Amalgamation of operational stressors subcategories.	Total count includes exposure to PPTE(s), sleep/shiftwork issues, hypervigilance, pain or injury, and issues with public.	113 (90)
Exposure to PPTE(s)	Exposure to one or more PPTE(s).	Reports of experiencing PPTE(s), including either singular events or cumulative exposures. Includes accounts of cumulative stress, compassion fatigue, and moral injury.	100 (79)
Sleep issues/shiftwork	Work/shiftwork causing issues with sleep.	Reduced sleep; nightmares or flashbacks while sleeping; adjusting to shift work; exhaustion; sleep difficulties.	89 (71)
Hypervigilance	Being in a state of constant alert, on edge, and feeling as if something bad is going to happen.	Being on edge from loud or sudden noises; hyper awareness about “bad people” in the world; nervousness and on edge in public places; isolating due to feeling on edge about personal safety in public; being “on alert” all the time.	62 (49)
Pain or injury	Pain or injury acquired through work.	Pain or injury leads to increase in symptoms, difficulties sleeping, challenges at work, and/or having to take short-term disability leave.	14 (11)
Issues with public	Stressors associated with dealing with the public.	Feeling disrespected or harassed by the public; current social movements advocating for defunding police; constant and increasing demands from public.	7 (6)
(2) Work impacting family life	Occupational stressors impact family life.	Communication issues; symptoms of withdrawal or increased lack of intimacy; lack of work/life balance; inadequate support for PSP work from spouse/partner; scheduling conflicts; vigilance regarding families’ safety.	64 (51)
(3) Organizational stressors	Amalgamation of organizational stressors subthemes.	Total count includes issues with leadership, resources and workload, issues with co-workers, and complaints.	57 (45)
Issues with administrators, leadership, or management	Endorsement of an unsupportive or toxic work environment created by superiors.	Feeling poorly treated (e.g., unsupported, unappreciated, or bullied) by superiors; belief that superiors are not protective of client’s safety, health, or wellbeing.	31 (25)
Resources and workload	Lack of resources as well as expectations related to workload.	Staff shortages; overcrowding in prison; lack of resources to meet the needs of the patients/clients they serve; general comments on lack of resources; managing increased call volumes; expectations for hours worked and overtime; inadequate compensation for expected workload; limited time off and breaks.	21 (17)
Issues with co-workers	Interpersonal conflicts or issues with co-workers/colleagues.	Bullying, harassment, and interpersonal conflict; toxic work environment created by colleagues; frustrations due to colleagues not meeting expectations or demands of the job.	17 (14)
Complaint	Complaints or disciplinary actions.	Disciplinary (or write up) complaint against the client; stress associated with filing a complaint against a co-worker or management.	5 (4)
(4) COVID-19-related work stress	Occupational stressors (both occupational and organizational) related to the COVID-19 pandemic.	Occupational stressors (both occupational and organizational) related to the COVID-19 pandemic.	55 (44)
(5) Unspecified occupational stress	Reports of work-related stress or work affecting symptoms without specifying the nature of the stress.	Reports of work-related stress or work affecting symptoms without specifying the nature of the stress.	34 (27)
**Personal Stressors**	Amalgamation of personal stressors.	Total count includes family concerns, PPTE(s), financial issues, previous mental health problems, other personal concerns, and personal health concerns.	83 (66)
(1) Family concerns	Concerns about immediate or extended family not reported as directly related to PSP work.	Spouse/partner experiencing stress from their own work, has substance abuse issues, or is experiencing low mood; death or illness of family member; family conflict; miscarriage; trust issues; financial issues; previous childhood issues impact current relationship; parenting concerns.	62 (49)
(2) PPTE(s)	Exposure to PPTE(s) in personal life.	Childhood trauma; sexual abuse or trauma; motor vehicle accident; victim of crime; family violence; sudden or unexpected violent death in family.	17 (14)
(3) Financial issues	Financial concerns.	Money mismanagement; debt; overspending; unexpected expenses.	15 (12)
(4) Previous mental health problems	Mental health issues prior to entering PSP work.	Previous mental health disorder diagnoses; symptoms experienced in prior to being a PSP, including in youth and childhood.	17 (14)
(5) Other personal concerns	Various stressors impacting mental health.	Feelings of loneliness; issues with religion; seasonal mood concerns.	10 (8)
(6) Personal health concerns	Injury or medical concern not related to occupational duties.	Hospitalizations; chronic health concerns; acute health concerns; physical health concerns.	9 (7)
**Proactive or education**	Accessed course as a proactive measure or for educational reasons.	Proactive/educational; experienced symptoms in the past or currently experiencing due to occupational stressors and want to mitigate symptom escalations. Increase knowledge as a means to provide peer-support or refer others to course.	10 (8)

Note. PPTE = potentially psychologically traumatic event. The table includes data from therapist screening notes for 126 clients. There were 32 clients out of the 126 who completed the telephone screen prior to the declaration of the COVID-19 pandemic in Saskatchewan. There were 86 clients who were directly asked about the impacts of COVID-19 during the telephone screen. There were 8 clients who completed the screen after the declaration of COVID-19, but were not directly screened for impacts.

**Table 4:** Occupational and personal issues shared with therapists.

Domain/Category	Definition	Examples	*n* (%)
**Occupational Stressors**	Amalgamation of occupational stressors categories.	Total count includes operational issues, organizational issues, symptom increases related to work, COVID-19 related, and work impacts on family life.	73 (58)
(1) Operational Stressors	Operational stressors increase symptoms or seeking advice on how to manage operational stressors.	PPTE(s); issues with the public; challenges with shiftwork; increased stress during occupational duties; high call volume; attending court/trial; pain or inability to perform work due to injury; sleep challenges.	42 (33)
(2) Organizational issues	Organizational stressors increase symptoms or seeking advice on how to manage organizational stressors.	Working short staffed; mistrust of or feeling unappreciated by managers; fears of disclosing mental health symptoms or reaching out for help; poor communication with colleagues/management; stress related to changes in positions.	35 (28)
(3) COVID-19-related	COVID-19 pandemic increased work stress or challenges.	Fears of bringing virus home; self-isolation due to exposure; staff shortages from illness and isolation; increasing call volume and demands from public; changing routines; stress over enforcing pandemic restrictions; feel health and safety is neglected by management; surges in the virus.	25 (20)
(4) Unspecified work stress	Work stress increasing symptoms without specifying nature of stressors.	Symptoms increase while at work or thinking about work; symptoms decrease while being away from work; thoughts about changing careers or regretting entering PSP work.	16 (13)
(5) Work impacts on family life	Discussions with therapist on how work is impacting family life.	Need to improve mental health to improve family life and relationships; need to improve work/life balance; realization that marriage issues are a result of cumulative trauma; managing shiftwork challenges; communication and anger issues with family; feeling a lack of support from family.	14 (11)
**Personal Stressors**	Amalgamation of themes under personal stressors or factors.	Total count includes family concerns, personal health or medication issues, other personal issues, and COVID-19-related issues.	49 (39)
(1) Family concerns	Immediate or extended family concerns not primarily related to work.	Spouse/partner thinking about switching careers; trust issues; impacted by spouse/partner’s low mood; stress from childcare and housework; pregnancy or miscarriage; spouse/partner or child’s physical or mental health condition; adjusting to moving in together with partner; stress over spouse/partner’s addictions; recent break up; family death or health issues; suicide threats by family; health of pet.	33 (26)
(2) Personal health concerns	Discussions of symptom changes or stressors associated with physical health problems or medications for mental health.	Side effects from mental health medication; accident/injury; medical problems; health issues from stress; weight loss issues.	22 (18)
(3) Other personal issues	Various personal stressors discussed with therapist.	Vehicle issues; feelings of having little purpose or direction.	7 (6)
(4) Financial	Financial concerns.	Consumer debt; costs of managing family’s health concerns and living arrangements; concerns about leaving job and finding a similar salary elsewhere.	*
(5) PPTE	PPTE experienced in personal life.	Reflections about childhood trauma.	*

Note. PPTE = potentially psychologically traumatic event. The table includes data from 126 clients. A total of 10 clients completed the course prior to the declaration of the COVID-19 pandemic in Saskatchewan. * Indicates there were at least 1, but fewer than 5, clients in the grouping. The exact number was masked to protect confidentiality.

**Table 5:** Skills that clients found helpful, found helpful or were working on without necessarily finding them helpful, or found challenging (*N* = 126).

Skills, *n,* (%)	Work-Related Context		Personal Context		No Specific Context		Any Context
	Found Skill Helpful	Found Skill Helpful or Working on	Found Skill Helpful	Found Skill Helpful or Working on	Found Skill Helpful	Found Skill Helpful or Working on	Challenges
(1) Thought challenging	21 (17)	27 (21)	15 (12)	18 (14)	59 (47)	74 (59)	39 (31)
(2) Recognizing cycle of symptoms	9 (7)	18 (14)	1 (1)	5 (4)	16 (13)	35 (28)	11 (9)
(3) Controlled breathing	11 (9)	14 (11)	4 (3)	4 (3)	24 (19)	37 (29)	8 (6)
(4) Graduated exposure	4 (3)	12 (9)	7 (6)	9 (7)	11 (9)	22 (18)	19 (15)
(5) Activity scheduling	3 (2)	4 (3)	4 (3)	6 (5)	16 (13)	34 (27)	12 (10)
(6) Relapse planning and goal setting	0	2 (1)	0	0	7 (6)	17 (14)	4 (3)

**Table 6:** Additional resources and PSP case stories that clients found helpful, found helpful or were working on without necessarily finding them helpful, or found challenging (*N* = 126).

Domain/Category	Found Resource Helpful	Found Resource Helpful or Working on	Challenges with Resource/Did Not Resonate with
**Additional resources, *n*, (%)**			
(1) PTSD	6 (4)	8 (4)	2 (2)
(2) Communication	5 (4)	9 (4)	2 (2)
(3) Worry	2 (2)	5 (3)	1 (1)
(4) Sleep	2 (2)	6 (3)	3 (2)
(5) Anger	4 (3)	6 (2)	1 (1)
(6) Structured problem solving	4 (3)	5 (2)	0
(7) Enhancing relationships	3 (2)	7 (5)	2 (2)
(8) Grief	2 (2)	3 (2)	0
(9) Managing beliefs	2 (2)	4 (2)	3 (2)
**Case stories and PSP examples, *n*, (%)**			
(1) Stories, examples, and PSP specific materials	47 (37)	NA	15 (11)

Note. PSP = public safety personnel. PTSD = post-traumatic stress disorder.

## Data Availability

Due to concerns regarding client confidentiality, the data used in this study will not be made available.

## Supplementary Materials

The following supporting information can be downloaded at: https://www.mdpi.com/article/10.3390/ijerph19084744/s1. Table S1: Occupational stressors reported at intake screen by gender, PSP occupation, and location of work; Table S2: Occupational stressors reported at intake screen by symptoms of mental disorders; Table S3: Occupational stressors discussed with therapist in client communication data by gender, PSP occupation, and location of work; Table S4: Occupational stressors discussed with therapist in client communication data by symptoms of mental disorders.

## Appendix A

Treatment Satisfaction Questionnaire

1. Would you feel confident recommending this treatment to a friend? Yes/No
2. Was it worth your time doing this course? Yes/No
3. Why or why not?
4. For you, what was the most helpful skill taught in this course? (For example, thought challenging, becoming more active, graded exposure, controlled breathing, other)
5. Can you give us an example of how a skill or strategy from the PSP Wellbeing Course made a difference in your life?
6. What did you like about the course? (For example, comments about the lessons, DIY Guides, Stories, or Resources)
7. What did you not like about the course? What should we do to improve it? (For example, comments about the lessons, DIY Guides, Stories, or Resources)
8. During the online course, have you experienced any unwanted negative events that you believe are related to the online treatment or have you encountered any unwanted negative effects that could be related to the online treatment?
9. If yes, please describe the unwanted negative events or unwanted negative effects, and define when during treatment these events/effects occurred, how often they happened, and how long they lasted. If you have experienced more than one event/effect, please describe each one separately.
