# Supplementary material for: Understanding and Addressing Occupational Stressors in Internet-Delivered Therapy for Public Safety Personnel: A Qualitative Analysis
Source: Int J Environ Res Public Health. 2022 Apr 14;19(8):4744. doi: 10.3390/ijerph19084744 (PMC9032164; doi:10.3390/ijerph19084744)
Supplement: Supplementary file 1 [file ijerph-19-04744-s001.zip › Supplementary Table S1.pdf]

Supplementary Table S1. Occupational stressors reported at intake screen by gender, PSP occupation, and location of work.

Domain/category	Gender		PSP occupation				Community size	
	Woman (*n* = 68)	Man (*n* = 57)	EMS (*n* = 38)	Police (*n* = 36)	Other (*n* = 32)	Corrections (*n* = 20)	Urban (*n* = 64)	Non-urban (*n* = 62)
I. Occupational stressors or factors, *n* (%)	65 (96)	57 (100)	36 (95)	35 (97)	31 (97)	20 (100)	63 (98)	59 (95)
i. Operational stressors	59 (87)	54 (95)	34 (89)	32 (89)	29 (91)	18 (90)	60 (94)	53 (85)
ii. Work impacting Family life	29 (43)	35 (61)	18 (47)	19 (53)	18 (56)	9 (45)	34 (53)	30 (48)
iii. Organizational stressors	36 (53)	21 (37)	17 (45)	16 (44)	14 (44)	10 (50)	24 (38)	33 (53)
iv. COVID-related work stress	26 (38)	29 (51)	13 (34)	15 (42)	15 (47)	12 (60)	20 (31)	14 (23)
v. Unspecified occupational stress	21 (31)	13 (23)	9 (24)	8 (22)	9 (28)	8 (40)	28 (44)	27 (44)

Note: The gender analysis excludes one participant who identified as non-binary to protect the confidentiality of the client. The category “Other” refers to PSP who identified the following occupations: border services, fire, dispatch/communications, and other.
