# Supplementary material for: Understanding and Addressing Occupational Stressors in Internet-Delivered Therapy for Public Safety Personnel: A Qualitative Analysis
Source: Int J Environ Res Public Health. 2022 Apr 14;19(8):4744. doi: 10.3390/ijerph19084744 (PMC9032164; doi:10.3390/ijerph19084744)
Supplement: Supplementary file 1 [file ijerph-19-04744-s001.zip › Supplementary Table S2.pdf]

Supplementary Table S2. Occupational stressors reported at intake screen by symptoms of mental disorders.

Domain/Category	PHQ-9		GAD-7		PCL-5	
	Clinically significant (n = 73)	Non-clinically significant (*n* = 53)	Clinically significant (n = 64)	Non-clinically significant (*n* = 60)	Clinically significant (n = 49)	Non-clinically significant (*n* = 77)
I. Occupational stressors or factors, *n* (%)	71 (97)	51 (96)	63 (98)	58 (97)	48 (98)	74 (97)
i. Operational stressors	68 (93)	45 (85)	62 (97)	50 (83)	47 (96)	66 (86)
ii. Work impacting Family life	42 (58)	22 (42)	35 (55)	29 (48)	28 (57)	36 (47)
iii. Organizational stressors	35 (48)	22 (42)	31 (48)	25 (42)	17 (35)	40 (52)
iv. COVID-related work stress	33 (45)	22 (42)	28 (44)	26 (43)	19 (39)	36 (47)
v. Unspecified occupational stress	23 (32)	11 (21)	23 (36)	11 (18)	11 (22)	23 (30)

Note: Two clients have missing data for the GAD-7.

GAD-7 = Generalized Anxiety Disorder-7 (anxiety); PHQ-9 = Patient Health Questionnaire-9 (depression); PCL-5 PTSD Checklist for DSM-5 (posttraumatic stress).
