# Supplementary material for: Understanding and Addressing Occupational Stressors in Internet-Delivered Therapy for Public Safety Personnel: A Qualitative Analysis
Source: Int J Environ Res Public Health. 2022 Apr 14;19(8):4744. doi: 10.3390/ijerph19084744 (PMC9032164; doi:10.3390/ijerph19084744)
Supplement: Supplementary file 1 [file ijerph-19-04744-s001.zip › Supplementary Table S3.pdf]

Supplementary Table S3. Occupational stressors discussed with therapist in client communication data by gender, PSP occupation, and location of work.

Domain/category	Gender		PSP occupation				Community size	
	Woman (n = 68)	Man (*n* = 57)	EMS (*n* = 38)	Police (*n* = 36)	Other (*n* = 32)	Corrections (*n* = 20)	Urban (*n* = 64)	Non-urban (*n* = 62)
I. Occupational stressors, *n* (%)	42 (62)	30 (53)	28 (74)	16 (44)	18 (56)	11 (55)	35 (55)	38 (61)
i. Operational issues	23 (34)	19 (33)	17 (45)	8 (22)	10 (31)	7 (35)	20 (31)	22 (35)
ii. Organizational issues	22 (32)	13 (23)	10 (26)	10 (28)	7 (22)	8 (40)	17 (27)	18 (29)
iii. COVID-19 related	13 (19)	11 (19)	10 (26)	3 (8)	6 (19)	6 (30)	14 (22)	11 (18)
iv. Unspecified occupational stress	9 (13)	7 (12)	6 (16)	5 (14)	2 (6)	3 (15)	6 (9)	10 (16)
v. Work-family conflict	10 (15)	4 (7)	1 (3)	6 (17)	5 (16)	2 (10)	7 (11)	7 (11)

Note: The gender analysis excludes one participant who identified as non-binary to protect the confidentiality of the client. The category "Other" refers to PSP who identified the following occupations: border services, fire, dispatch/communications, and other.
