# Supplementary material for: Understanding and Addressing Occupational Stressors in Internet-Delivered Therapy for Public Safety Personnel: A Qualitative Analysis
Source: Int J Environ Res Public Health. 2022 Apr 14;19(8):4744. doi: 10.3390/ijerph19084744 (PMC9032164; doi:10.3390/ijerph19084744)
Supplement: Supplementary file 1 [file ijerph-19-04744-s001.zip › Supplementary Table S4.pdf]

Supplementary Table S4. Occupational stressors discussed with therapist in client communication data by symptoms of mental disorders.

Domain/Category	PHQ-9		GAD-7		PCL-5	
	Clinically significant (n = 73)	Non-clinically significant (*n* = 53)	Clinically significant (n = 64)	Non-clinically significant (*n* = 60)	Clinically significant (n = 49)	Non-clinically significant (*n* = 77)
I. Occupational stressors, *n* (%)	45 (62)	28 (53)	41 (64)	31 (52)	32 (65)	41 (53)
i. Operational issues	27 (37)	15 (28)	24 (38)	17 (28)	21 (43)	21 (27)
ii. Organizational issues	22 (30)	13 (25)	20 (31)	15 (25)	12 (24)	23 (30)
iii. COVID-19 related	15 (21)	10 (19)	15 (23)	10 (17)	12 (24)	13 (17)
iv. Unspecified occupational stress	12 (16)	4 (8)	12 (19)	4 (7)	5 (10)	11 (14)
v. Work-family conflict	9 (12)	5 (9)	7 (11)	7 (12)	5 (10)	9 (12)

Note: Two clients have missing data for the GAD-7.
GAD-7 = Generalized Anxiety Disorder-7 (anxiety); PHQ-9 = Patient Health Questionnaire-9 (depression); PCL-5 PTSD Checklist for DSM-5 (posttraumatic stress).
